# Novel Type V-A CRISPR Effectors Are Active Nucleases with Expanded Targeting Capabilities

**DOI:** 10.1089/crispr.2020.0043

**Published:** 2020-12-17

**Authors:** Daniela S. Aliaga Goltsman, Lisa M. Alexander, Audra E. Devoto, Justine B. Albers, Jason Liu, Cristina N. Butterfield, Christopher T. Brown, Brian C. Thomas

**Affiliations:** Metagenomi, Inc., Emeryville, California, USA.

## Abstract

Cas12a enzymes are quickly being adopted for use in a variety of genome-editing applications. These programmable nucleases are part of adaptive microbial immune systems, the natural diversity of which has been largely unexplored. Here, we identified novel families of Type V-A CRISPR nucleases through a large-scale analysis of metagenomes collected from a variety of complex environments, and developed representatives of these systems into gene-editing platforms. The nucleases display extensive protein variation and can be programmed by a single-guide RNA with specific motifs. The majority of these enzymes are part of systems recovered from uncultivated organisms, some of which also encode a divergent Type V effector. Biochemical analysis uncovered unexpected protospacer adjacent motif diversity, indicating that these systems will facilitate a variety of genome-engineering applications. The simplicity of guide sequences and activity in human cell lines suggest utility in gene and cell therapies.

## Introduction

CRISPR-Cas systems have emerged in recent years as the gene-editing technology of choice due to their targetability and ease of use.^1^ The most commonly used enzymes are the Class 2 Type II SpCas9^2^ and the Class 2 Type V-A Cas12a (previously Cpf1) effectors.^3^ The Cas12a nucleases in particular are becoming more widely used, since their reported specificity in cells is higher than other nucleases, with fewer or no detectable off-target effects.^4,5^ The V-A nucleases are also advantageous in that the guide RNA is small (42–44 nucleotides compared to approximately 100 nucleotides for SpCas9) and is processed by the nuclease itself following transcription from the CRISPR array, simplifying multiplexed applications with multiple gene edits.^6^ Furthermore, the V-A nucleases have staggered cut sites, which may facilitate directed repair pathways, such as microhomology-dependent targeted integration.^7^

The most commonly used Cas12a nucleases require a 5′ protospacer adjacent motif (PAM) next to the chosen target site: 5′-TTTV-3′ for *Lachnospiraceae* bacterium ND2006 LbCas12a and *Acidaminococcus sp.* AsCas12a; and 5′-TTV-3′ for *Francisella novicida* FnCas12a.^3^ Recent exploration of orthologs has revealed proteins with less restrictive PAM sequences that are also active in mammalian cell culture, for example YTV, YYN, or TTN.^8–11^ However, these Cas12a orthologs do not fully encompass the biodiversity of these systems, and therefore likely do not represent all possible activities and PAM sequence requirements. Here, we mined thousands of genomic fragments from numerous metagenomes for Cas12a nucleases. We sought to expand the known diversity of these enzymes and develop novel systems into highly targetable, compact, and precise gene-editing agents.

## Methods

Thirteen animal microbiome, high-temperature biofilm, and sediment samples were collected and stored on ice or in Zymo DNA/RNA Shield after collection. DNA was extracted from samples using either the Qiagen DNeasy PowerSoil Kit or the ZymoBIOMICS DNA Miniprep Kit. DNA sequencing libraries were constructed (Illumina TruSeq) and sequenced on an Illumina HiSeq 4000 or Novaseq at the Vincent J. Coates Genomics Sequencing Laboratory at UC Berkeley, with paired 150 bp reads with a 400–800 bp target insert size (10 gb of sequencing was targeted per sample; Supplementary Table S1). Publicly available metagenomic sequencing data were downloaded from the National Center for Biotechnology Information Sequence Read Archive (Supplementary Table S1). Sequencing reads were trimmed using BBMap (Bushnell B., sourceforge.net/projects/bbmap/) and assembled with Megahit.^12^ Open reading frames and protein sequences were predicted with Prodigal.^13^ HMM profiles of known Type V-A CRISPR nucleases were built and searched against all predicted proteins using HMMER3 (hmmer.org) to identify potential Cas12a effectors. CRISPR arrays were predicted on assembled contigs with Minced (https://github.com/ctSkennerton/minced).^14^ Taxonomy was assigned to proteins with Kaiju,^15^ and contig taxonomy was determined by finding the consensus of all encoded proteins.

Predicted and reference (e.g., LbCas12a, AsCas12a, and FnCas12a) Type V-A effector proteins were aligned with MAFFT^16^ (Supplementary File S1), and a phylogenetic tree was inferred using FastTree2^17^ (Supplementary File S2). Novel families were delineated by identifying clades composed of sequences recovered from this study. From within families, candidates were selected if they contained all necessary components for laboratory analysis (i.e., they were found on a well-assembled and annotated contig with a CRISPR array) in a manner that sampled as much phylogenetic diversity as possible. Priority was given to small effectors from diverse families (i.e., families with representatives sharing a wider range of protein sequences). Selected representative and reference sequences were aligned using MUSCLE^18^ and Clustal W^19^ to identify catalytic and PAM interacting residues. CRISPR array repeats were searched for a motif associated with Cas12a nucleases, TCTAC-N-GTAGA (containing between one and eight N residues). From this analysis, families were putatively classified as V-A if representative CRISPR arrays contained one or more of these motif sequences. This data set was used to identify HMM profiles associated with V-A families, which were in turn used to classify additional families. Protein and single-guide RNA (sgRNA) sequences are available in Supplementary Table S2. Although the convention is to name novel Cas12a nucleases on the basis of the organism that encodes them, it is not possible to do so accurately in cases when the strains are not yet characterized. Therefore, in order to adhere to the convention best, we have named these proteins with a suffix MX-Y, where M indicates that the proteins are derived from assembled metagenomic fragments, X represents the family identifier, and Y indicates the member identifier. For example Cas12a-M29-1, a Cas12a enzyme recovered from metagenomics data, is the first member of family 29.

*In vitro* nuclease cleavage activity was tested, and PAM requirements were determined via an *Escherichia coli* lysate-based expression system (myTXTL; Arbor Biosciences), with modifications.^20–22^ Briefly, the *E. coli* codon optimized protein sequences were expressed under control of a T7 promoter at 29°C for 16 h. This crude protein stock was then used in an *in vitro* digest reaction at a concentration of 20% of the final reaction volume. The reaction was incubated for 3 h at 37°C with 5 nM of a plasmid library consisting of a constant spacer sequence preceded by 8N mixed bases, and 50 nM of *in vitro* transcribed crRNA targeting to the spacer in NEB buffer 2.1 (New England Biolabs; NEB buffer 2.1 was selected in order to compare candidates with commercially available proteins). Protein concentration was not normalized in PAM discovery assays (polymerase chain reaction amplification signal provides high sensitivity for low expression or activity).

The cleavage products from the TXTL reactions were recovered via cleanup with AMPure SPRI beads (Beckman Coulter). The DNA was blunted via addition of Klenow fragments and dNTPs (New England Biolabs). Blunt-end products were ligated with a 100-fold excess of double-stranded adapter sequences, and used as template for the preparation of a next-generation sequencing (NGS) library, from which PAM requirements were determined from sequence analysis.

Raw NGS reads were filtered by Phred quality score >20. The 28 bp representing the known DNA sequence from the backbone adjacent to the PAM was used as a reference to find the PAM-proximal region, and the 8 bp adjacent was identified as the putative PAM. The distance between the PAM and the ligated adapter was also measured for each read. Reads that did not have an exact match to the reference sequence or adapter sequence were excluded. PAM sequences were filtered by cut site frequency such that only PAMs with the most frequent cut site ±2 bp were included in the analysis. This correction removes low levels of background cleavage that may occur at random positions due to the use of crude *E. coli* lysate. This filtering step can remove between 2% and 40% of the reads, depending on the signal-to-noise ratio of the candidate protein, where less active proteins have more background signal. For reference, for Cas12a-M29-1, 2% of reads were filtered out at this step. The filtered list of PAMs was used to generate a sequence logo using Logomaker.^23^

Active candidates were tested for cleavage activity in HEK293T cells. Briefly, the protein sequences were cloned into an expression vector with N- and C-terminal SV40 nuclear localization signals (NLSs), and a 2A-GFP tag after the C-terminal NLS. The sgRNA sequence was cloned into a separate expression vector. HEK293T cells were then co-transfected with 140 ng nuclease plasmid and 60 ng sgRNA plasmid. After 72 h, the DNA was extracted and used for NGS sequencing. At least 10 different target loci were chosen for testing the activity of each protein. The target site sequences in human cells and the primers used to amplify these sites for NGS are available in Supplementary Table S3.

Genome-editing efficiency in human cells was assessed from the NGS reads with CRISPResso^24^ using the following parameters: cleavage offset = −4 and window = 10. All post-cleavage events from the CRISPResso output were summed for ±1 bp indels/mutations and ≥2 bp deletions, insertions, and mutations. All outcomes were normalized to total sequences aligned to the expected amplicon. Ribonucleoprotein (RNP) and plasmid-editing profile data for Cas12a-M29-1 and AsCas12a are included in Supplementary Table S4.

## Results

### Novel effectors

**FIG. 1.**
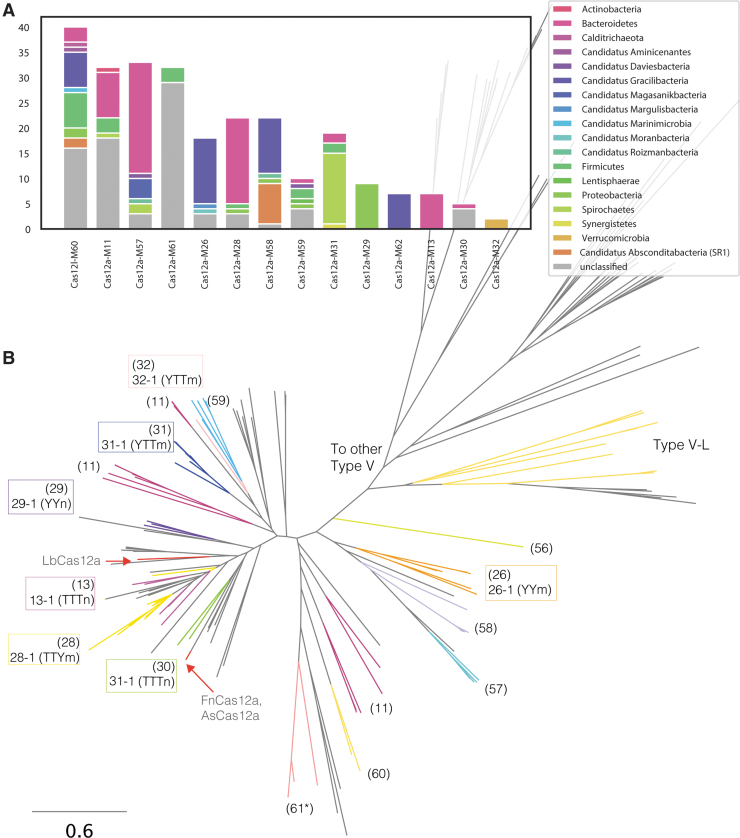
Diversity of CRISPR Type V-A effectors. **(A)** Per family distribution of taxonomic classification of contigs encoding the novel Type V-A and V-L effectors. **(B)** Phylogenetic tree inferred from an alignment of 119 novel Type V-A and V-L (colored branches) and 89 reference (gray branches) sequences. Cas12a-M families are denoted by numbers in parentheses. Protospacer adjacent motif (PAM) requirements for active nucleases described here are outlined within boxes colored by family. Non-Type V-A reference sequences were used to root the tree. *Cas12a-M61 family requires a crRNA with an alternative repeat motif.

We mined 140,867 Mbp of assembled metagenomic sequencing data from diverse environments (soil, thermophilic, sediments, and human and nonhuman microbiomes; Supplementary Fig. S1A and Supplementary Table S1). In total, 119 genomic fragments contained effectors distantly related to Cas12a nucleases encoded next to a CRISPR array (Fig. 1, Supplementary Table S2, and Supplementary File S1). These Type V-A effectors were classified into 14 novel families sharing <30% average pairwise amino acid identity between each other, and with reference sequences (e.g., LbCas12a, AsCas12a, and FnCas12a). Some effectors contained RuvC and alpha-helical recognition domains, as well as conserved DED nuclease catalytic residues (identified in multiple sequence alignments), suggesting that these effectors are active nucleases (Supplementary Figs. S1–S3). The novel Cas12a nucleases range between 1,000 and 1,400 aa in length (Supplementary Fig. S1A–C, Supplementary Table S2, and Supplementary File S1), and the taxonomic classifications of the contigs that encode them span a diverse array of phyla (Fig. 1).

Some genomic fragments carrying a Type V-A CRISPR system also encoded a distant Type V effector, referred to here as Type V-L (Fig. 1). For example, Cas12l-M60-9 shares only 16.6% amino acid identity with the Type V-A Cas12a-M26-1. Both effectors are encoded in the same CRISPR Cas operon, and likely share the same crRNA with Cas12a-M26-1 (Supplementary Fig. S3). Although no nuclease domains were predicted, Cas12l-M60-9 contains three RuvC catalytic residues identified from multiple sequence alignments and 3D structure prediction (Supplementary Fig. S3).

### RNA guide identification

For contigs that encoded a Cas12a effector and a CRISPR array, secondary structure folding of repeats indicate that the novel Cas12a nucleases require a sgRNA (Supplementary Fig. S4 and Supplementary Table S3). No tracrRNA sequences could be reliably predicted. The sgRNA consists of ∼19–22 bp from the 3′ end of the CRISPR repeat. A multiple sequence alignment of CRISPR repeats from seven of the Cas12a candidates tested for *in vitro* activity indicates a highly conserved motif at the 3′ end of the repeat, which forms the stem–loop structure of the sgRNA (Supplementary Fig. S4). The motif, UCUAC[N3-5]GUAGAU, consists of short palindromic repeats (the stem) separated by between three and five nucleotides (the loop).

We sought to use the conservation of the sgRNA motif to uncover novel effectors that may not show similarity to classified Cas12a nucleases. Motifs were searched in repeats from 69,117 CRISPR arrays. The most common motif contained a four-nucleotide loop, while three- and five-nucleotide loops are less common (Supplementary Fig. S1D). Inspection of the genomic context surrounding the CRISPR arrays containing the repeat motif revealed numerous effectors of varying lengths. For example, effectors of the family Cas12a-M57 are the largest of the Cas12a nucleases identified (average ∼1,400 aa) and encode a repeat with a 4 bp loop. Two other families identified from HMM analysis contained a rare repeat motif, CCUGC[N4]GCAGG (Cas12a-M11 and Cas12a-M61; Fig. 1). Although differing in sequence, the structure is predicted to fold into a stem–loop structure similar to the common V-A repeat (Supplementary Fig. S4C and D).

### *In vitro* activity

Promising candidates from bioinformatic analysis were selected for biochemical screening. Using the conserved 3′ sgRNA structure, a “universal” sgRNA was designed comprising the 3′ 20 nucleotides of the CRISPR repeat and a 24-nucleotide spacer (Supplementary Fig. S4 and Supplementary Table S2). Of the seven tested candidates, six showed activity *in vitro* against the 8N PAM library (Supplementary Fig. S5A). The remaining inactive candidate (Cas12a-M30-1) showed activity when tested with its endogenous trimmed CRISPR repeat but was not included in NGS library assays (Supplementary Fig. S5B).

**FIG. 2.**
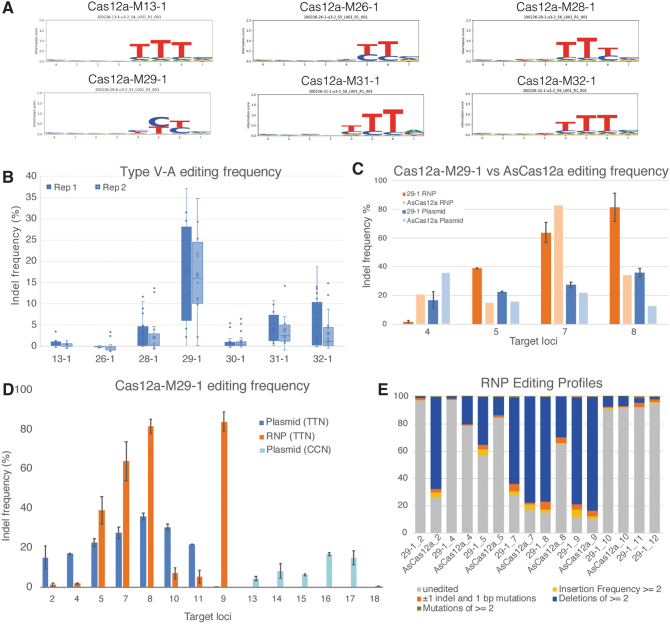
Type V-A effectors are active nucleases. **(A)** PAM SeqLogo determined for six Type V-A nucleases. **(B)** Boxplot of plasmid transfection activity assays inferred from frequency of indel edits for active nucleases. The boundaries of the boxplots indicate first and third quartile values. The mean is indicated with an “x” and the median is represented by the midline within each box. **(C)** Plasmid transfection editing frequencies at four target sites for Cas12a-M29-1 versus AsCpf1. Side-by-side comparisons represent one experiment. **(D)** Plasmid versus ribonucleoprotein (RNP) editing activity for nuclease Cas12a-M29-1 at 14 target loci. **(E)** Editing profile of nuclease Cas12a-M29-1 from RNP transfection assays. Editing frequency experiments in **(B)**, **(D)**, and **(E)** were done in duplicate. The bar plots in **(C)** and **(D)** show mean editing frequency with one standard deviation error bars **(D)**.

The majority of identified PAMs are thymine-rich sequences of two or three bases (Fig. 2A). However, two enzymes—Cas12a-M26-1 (PAM YYN) and Cas12a-M29-1 (PAM YYN)—have PAM specificity for pyrimidine base, thymine, or cytosine, allowing for broader sequence targeting. Analysis of putative PAM-interacting residues indicates that the active Cas12a nucleases, as well as many non-tested effectors, contain a conserved lysine and a GWxxxK motif (Supplementary Fig. S2), which may be important in recognition and interaction with different PAMs as shown in LbCas12a.^25^

The requirement for end repair to create blunt-end fragments prior to ligation in the PAM enrichment protocol suggests that these enzymes create a staggered double-strand DNA break, similar to previously reported Cas12a nucleases. The cut site on the target strand was identified to occur at the 22nd PAM distal base by analysis of the NGS reads (Supplementary Fig. S5C).

### Genome editing

After confirmation of the PAM, the novel V-A effectors were tested in HEK293T cells for gene-targeting activity. All candidates showed activity >5% non-homologous end joining (NHEJ; background corrected) on plasmid transfections on at least 1/10 tested target loci (Fig. 2B). Cas12a-M29-1 showed the highest overall activity in both NHEJ modification outcomes and activity across different targets (Fig. 2B–D). Preliminary side-by-side plasmid transfection assays indicate that Cas12a-M29-1 has higher editing efficiency than AsCas12a at three of four target loci (Fig. 2C). Furthermore, Cas12a-M29-1 showed above background editing activity on plasmid transfection assays for five out of nine guides with CCN PAMs, although efficiency is lower compared to its preferred TTN PAMs (Fig. 2D). Therefore, this nuclease was selected for purified RNP complex testing in HEK293 cells. RNP transfection of Cas12a-M29-1 holoenzyme showed higher editing levels with RNP than plasmid-based transfection on four out of eight targets, in some cases >80% editing efficiency (Fig. 2D). Analysis of editing profiles for Cas12a-M29-1 indicates that this nuclease produces deletions ≥2 bp more frequently than other types of edits at their target site (Fig. 2E). Notably, Cas12a-M29-1 editing via RNP at the same target as AsCpf1 shows more than twice the efficiency of the control at two target sites (Fig. 2E and Supplementary Fig. S6).

## Discussion

We conducted a broad analysis of novel Type V-A CRISPR families based on analysis of metagenomes collected from a variety of complex environments. These novel Cas12a nucleases are diverse within and across families, require a sgRNA, and show different targeting ability by recognizing different PAMs. Similar to LbCas12a, AsCas12a, and FnCas12a, these novel type V-A effectors utilize crRNA for guided staggered double-stranded cleavage of DNA, simplifying guide design and synthesis, and enabling simplified multiplexed editing.^6^ Analysis of CRISPR repeat motifs that form the stem–loop structure of the crRNA suggests that most of the novel Type V-A effectors use a guide with a 4 bp loop more frequently than shorter or longer loops. The sgRNA motif from LbCas12a uses a less common 5 bp loop, although the 4 bp loop was also observed previously for 16 Cpf1 orthologs.^3^ A rare CRISPR repeat motif that folds into a stem–loop sgRNA was also predicted for two novel families of Type V-A effectors. The high degree of conservation of the sgRNA with variable loop lengths in Type V-A may afford flexible levels of activity, as shown for one of our novel candidates. Taken together, these effectors are not close homologs to previously studied enzymes, and greatly expand the diversity of Cas12a sgRNA nucleases.

We further identified novel Type V effectors, some of which are encoded next to Cas12a nucleases. In some cases, both nucleases share a CRISPR sgRNA, but the novel effectors are divergent from Cas12a (Fig. 1). For the effectors encoded in CRISPR operons without a Type V-A nuclease, the CRISPR repeat also folds into a sgRNA with the V-A UCUAC[N3-5]GUAGAU motif. We predict that the Cas12a and the novel Type V nucleases arose from an ancient duplication event followed by independent evolution. Based on the presence of catalytic residues, we predict that these novel effectors are active nucleases. One report identified a Type V effector, referred to by the authors as cms1, encoded next to a Type V-A nuclease, which requires a sgRNA for cleavage activity in plant cells.^26^ The authors report different CRISPR arrays for each effector, while the novel Type V system described here suggests that both nucleases may use the same crRNA for DNA targeting and cleavage. As described recently in Roizmanbacteria genomes,^27^ both Cas12a and the associated Type V effectors are distantly related based on sequence similarity and phylogenetic analysis. Therefore, the novel effectors described here likely do not belong within the Type V-A classification. Following current nomenclature of Type V CRISPR systems,^28–32^ we propose a separate Type V subclassification and name: Type V-L CRISPR systems.

PAMs determined for active Cas12a-M family nucleases are generally thymine rich, similar to other Type V-A proteins that have been reported.^3^ However, a shorter YYN PAM sequence observed for Cas12a-M29-1 increases the target flexibility compared to the TTTV PAM of LbCas12a, much like the proteins from *Moraxella bovoculi* isolates.^8^ Additional experiments are needed on other Cas12a family members to draw broader conclusions about PAM specificity across diverse clades of sequences.

When testing the novel nucleases for *in vitro* editing activity, Cas12a-M29-1 levels compare to plasmid transfections reported elsewhere.^3^ Reports of plasmid transfection editing efficiencies in Cas12a orthologs indicate between 21% and 26% indel frequencies for guides with T-rich PAMs, and 1/18 guides with CCN PAMs showed ∼10% activity in Mb3Cas12a.^8^ Notably, Cas12a-M29-1 activity in plasmid transfections appears greater than that reported for Mb3Cas12a for targets with TTN and CCN PAMs. Because the target sites for plasmid transfections have the same TTG PAM on all experiments, the difference in editing efficiency may be attributed to genomic accessibility differences at different target genes. Cas12a-M29-1 editing as RNP is much more efficient than via plasmid and is more efficient than AsCas12a on two of seven target loci. Therefore, Cas12a-M29-1 is a highly active and efficient gene-editing nuclease.

## Conclusion

The findings presented here increase the known diversity of single guide Type V-A CRISPR nucleases, and demonstrate the genome editing potential of novel enzymes from uncultivated microbes. Seven novel nucleases showed *in vitro* activity with diverse PAM requirements, and RNP data showed editing efficiency >80% for therapeutically relevant targets in human cell lines. We anticipate that these novel nucleases expand the toolkit of CRISPR-associated enzymes and will enable diverse genome-engineering applications.

## Supplementary Material

Supplemental data

Supplemental data

Supplemental data

Supplemental data

Supplemental data

Supplemental data

Supplemental data

Supplemental data

Supplemental data

Supplemental data

Supplemental data

Supplemental data
