# Supplementary material for: Novel Type V-A CRISPR Effectors Are Active Nucleases with Expanded Targeting Capabilities
Source: CRISPR J. 2020 Dec 17;3(6):454–61. doi: 10.1089/crispr.2020.0043 (PMC7757703; doi:10.1089/crispr.2020.0043)

Supplementary Figure 1. Per family distribution of effector protein length by type of sample (A), presence of RuvC catalytic residues (B), and type of repeat motif for their associated CRISPR array (C). D) Distribution of the type of repeat motifs commonly associated with Cas12a effectors.


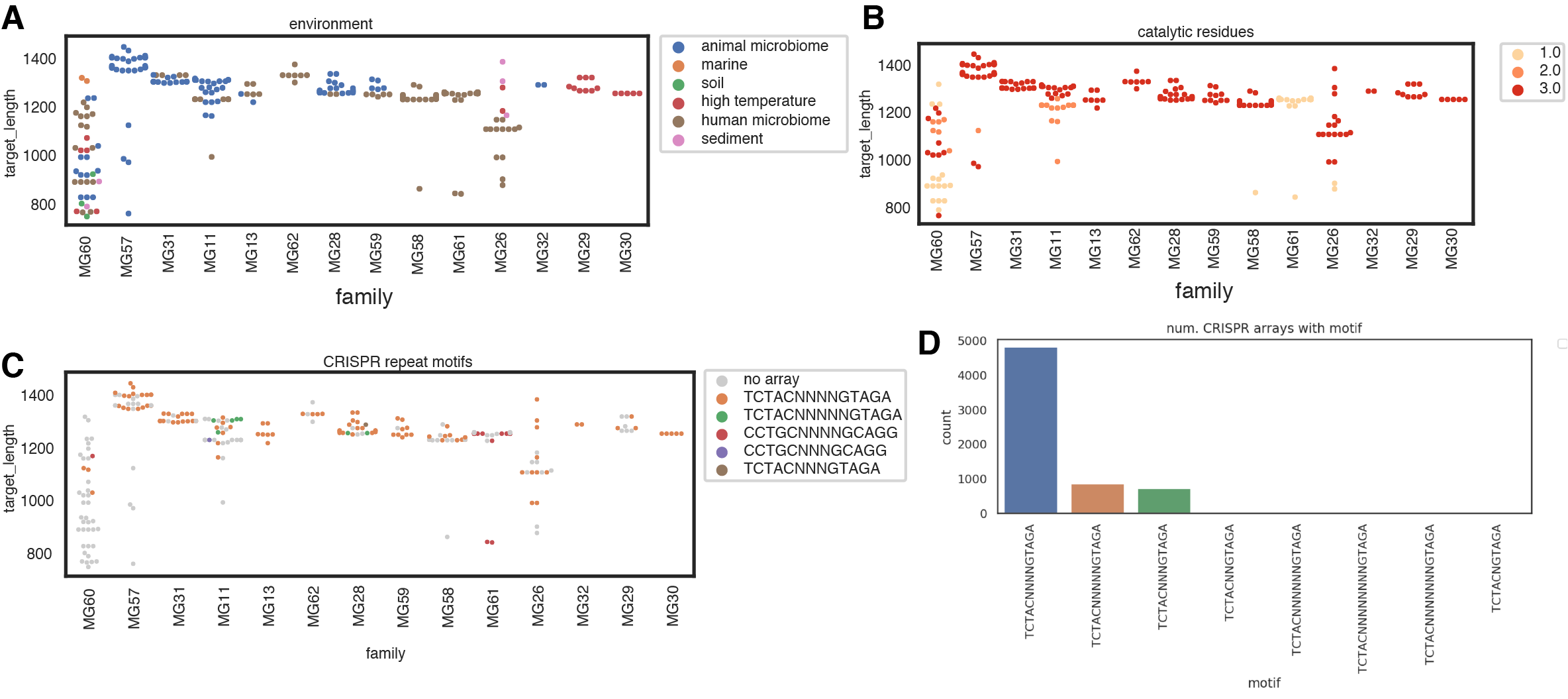

Supplement: Supplemental data [file Supp_Fig1.docx]
