# Supplementary material for: Novel Type V-A CRISPR Effectors Are Active Nucleases with Expanded Targeting Capabilities
Source: CRISPR J. 2020 Dec 17;3(6):454–61. doi: 10.1089/crispr.2020.0043 (PMC7757703; doi:10.1089/crispr.2020.0043)

Supplementary Figure 3. Analysis of co-occurring Type V-A and V-L effectors. A) Genomic context of Cas12a-M26-1 and Cas12l-M60-9 effectors in a CRISPR operon. Type V effectors are indicated by orange arrows. The CRISPR array is indicated by a blue bar. Predicted domains for each protein in the contig are indicated by pink boxes. B) Multiple sequence alignment of Type V-L Cas12l-M60-9 and AsCas12a reference sequence. Top: alignment shows the identified RuvC catalytic residues (11.1% pairwise aa identity, catalytic residues are indicated by blue squares). Middle: region containing the RuvC-I and RuvC-II catalytic residues. Bottom: region containing the RuvC-III catalytic residue.


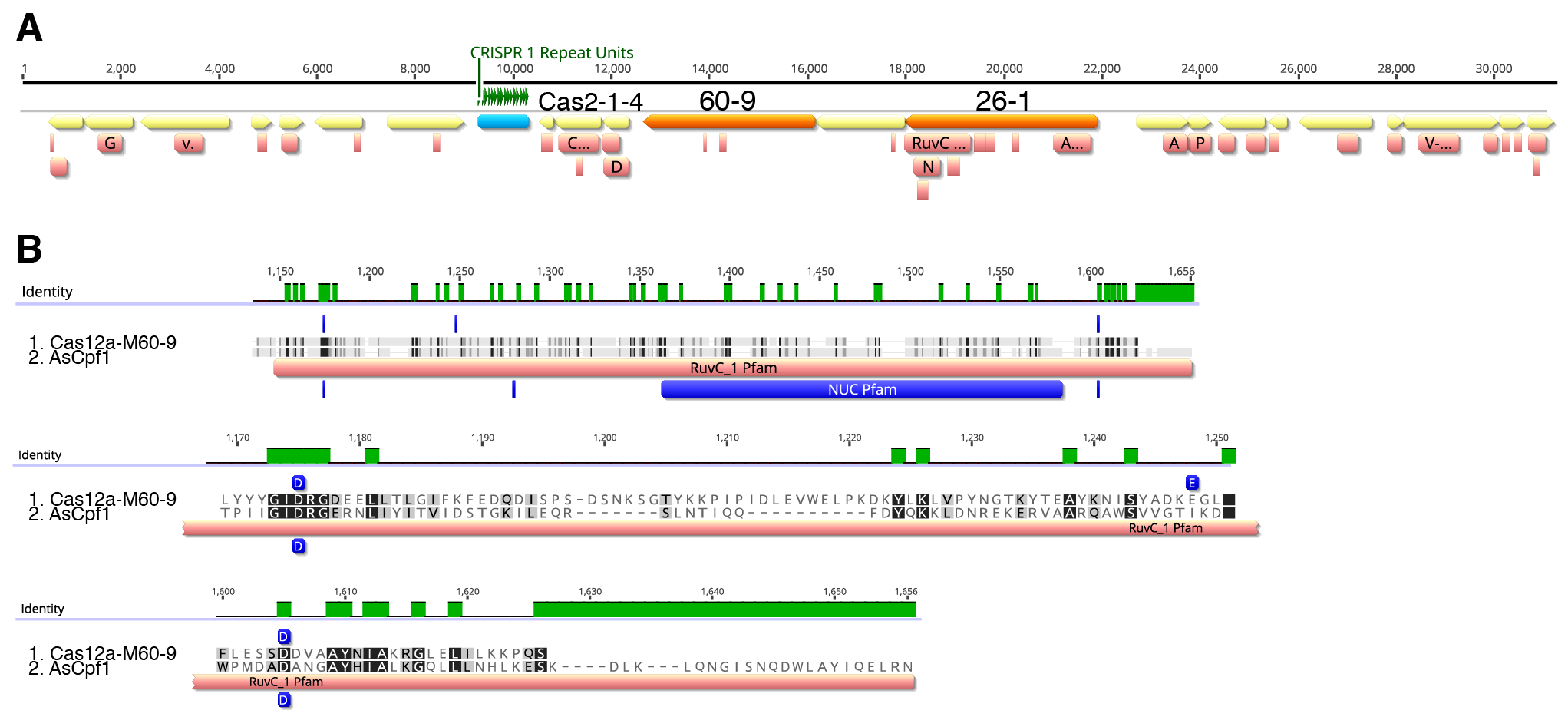

Supplement: Supplemental data [file Supp_Fig3.docx]
