# Supplementary material for: Novel Type V-A CRISPR Effectors Are Active Nucleases with Expanded Targeting Capabilities
Source: CRISPR J. 2020 Dec 17;3(6):454–61. doi: 10.1089/crispr.2020.0043 (PMC7757703; doi:10.1089/crispr.2020.0043)

Supplementary Figure 4. The CRISPR RNA (crRNA) structure is conserved among Type V-A systems. A) Fold structure of the reference crRNA sequence in the LbCpf1 system. B) Multiple sequence alignment of CRISPR repeats associated with novel Type V-A systems. The LbCpf1 processing site is indicated with a pink bar. C) Fold structure of Cas12a-M61-2 putative crRNA with a novel stem-loop motif CCUGC[N4]GCAGG. D) Multiple sequence alignment of CRISPR repeats with the novel repeat motif.


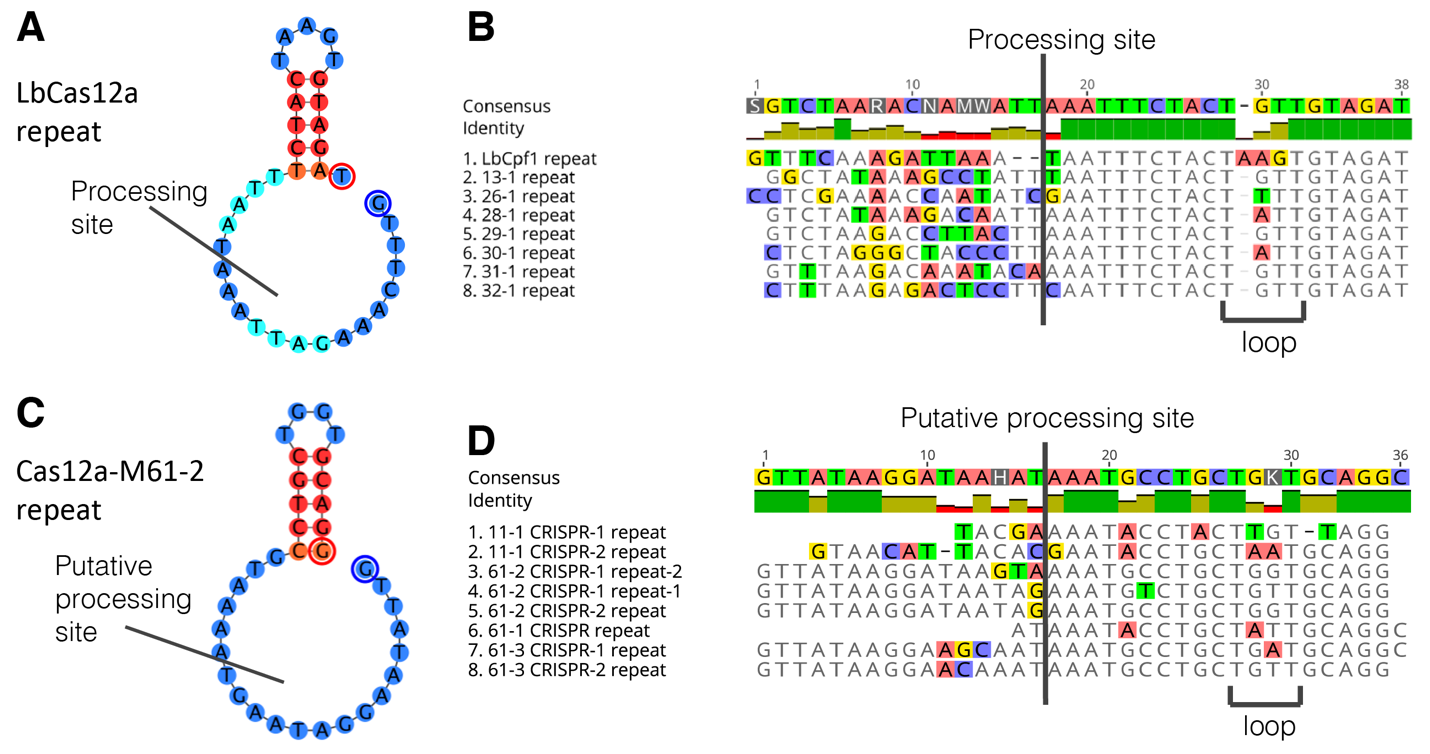

Supplement: Supplemental data [file Supp_Fig4.docx]
