# Supplementary material for: Novel Type V-A CRISPR Effectors Are Active Nucleases with Expanded Targeting Capabilities
Source: CRISPR J. 2020 Dec 17;3(6):454–61. doi: 10.1089/crispr.2020.0043 (PMC7757703; doi:10.1089/crispr.2020.0043)

Supplementary Figure 5. Biochemical characterization of Type V-A nucleases. A) PCR gels of the ligation products show activity of candidates Cas12a-M13-1, M26-1, M28-1, M29-1, M31-1 and M32-1 and of Cpf1 (positive control). Cleavage product band labeled with a red arrow. B) PCR gels of the ligation products confirm that Cas12a-M30-1 is active with its native crRNA versus using the “universal” sequence. Cleavage product band indicated with a red arrow. C) Analysis of the NGS cut sites shows cleavage on the target strand at position 22, sometimes with less frequent cleavage after 21 or 23 nt.


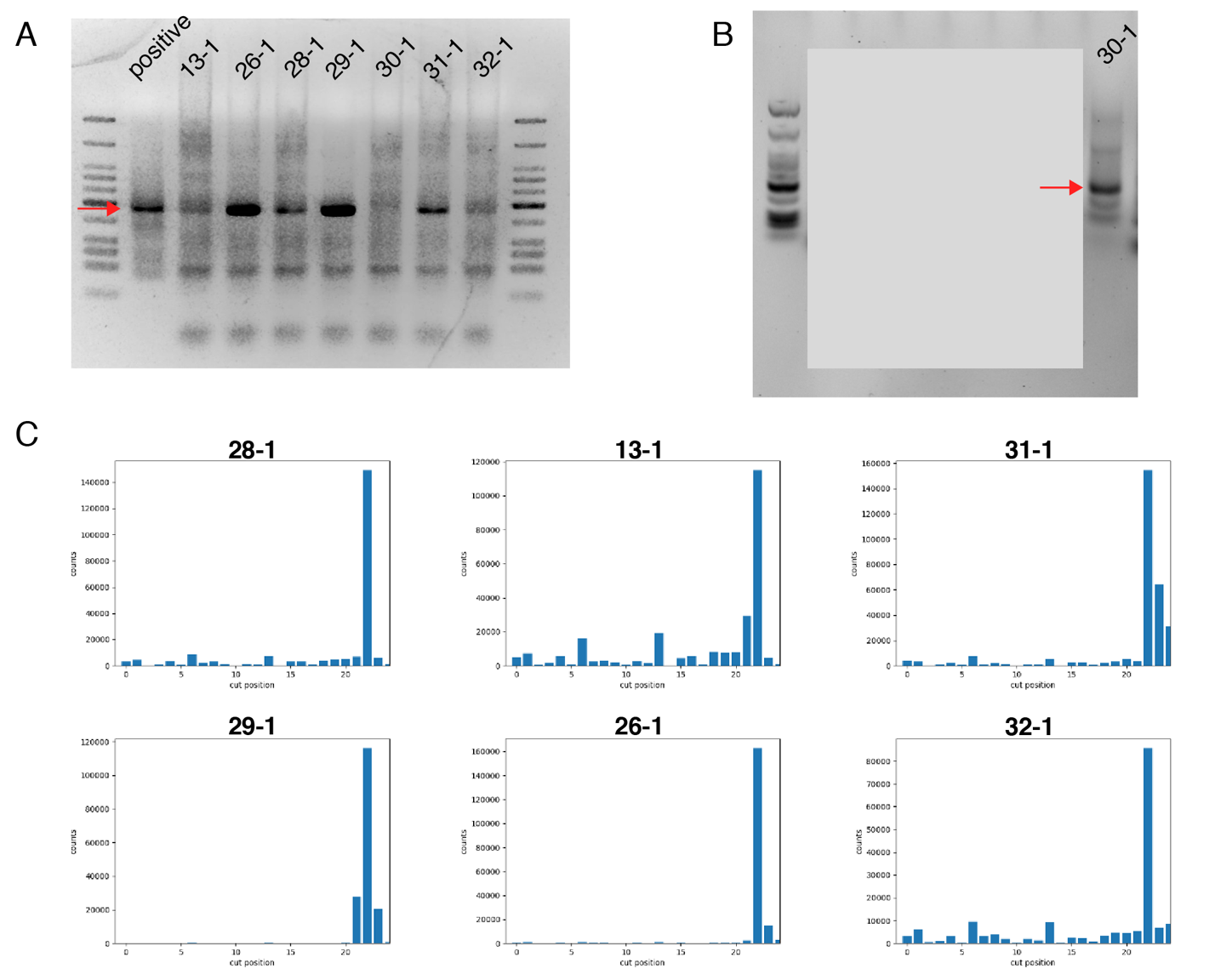

Supplement: Supplemental data [file Supp_Fig5.docx]
