# Supplementary material for: Novel Type V-A CRISPR Effectors Are Active Nucleases with Expanded Targeting Capabilities
Source: CRISPR J. 2020 Dec 17;3(6):454–61. doi: 10.1089/crispr.2020.0043 (PMC7757703; doi:10.1089/crispr.2020.0043)

Supplementary Figure 2. Multiple sequence alignment of catalytic and PAM interacting regions in novel Cas12a sequences. A) Blocks of conservation around the DED catalytic residues in RuvC-I (left), RuvC-II (middle), and RuvC-III (right) regions. B) WED-II and PAM interacting regions containing residues involved in PAM recognition and interaction in the reference sequence from *Francisella novicida* Cas12a. Other references included are *Acidaminococcus* sp. (AsCas12a), *Moraxella bovoculi* (MbCas12a), and *Lachnospiraceae* bacterium ND2006 (LbCas12a). RuvC catalytic and PAM interacting residues are highlighted with blue boxes on the reference FnCas12a sequence.


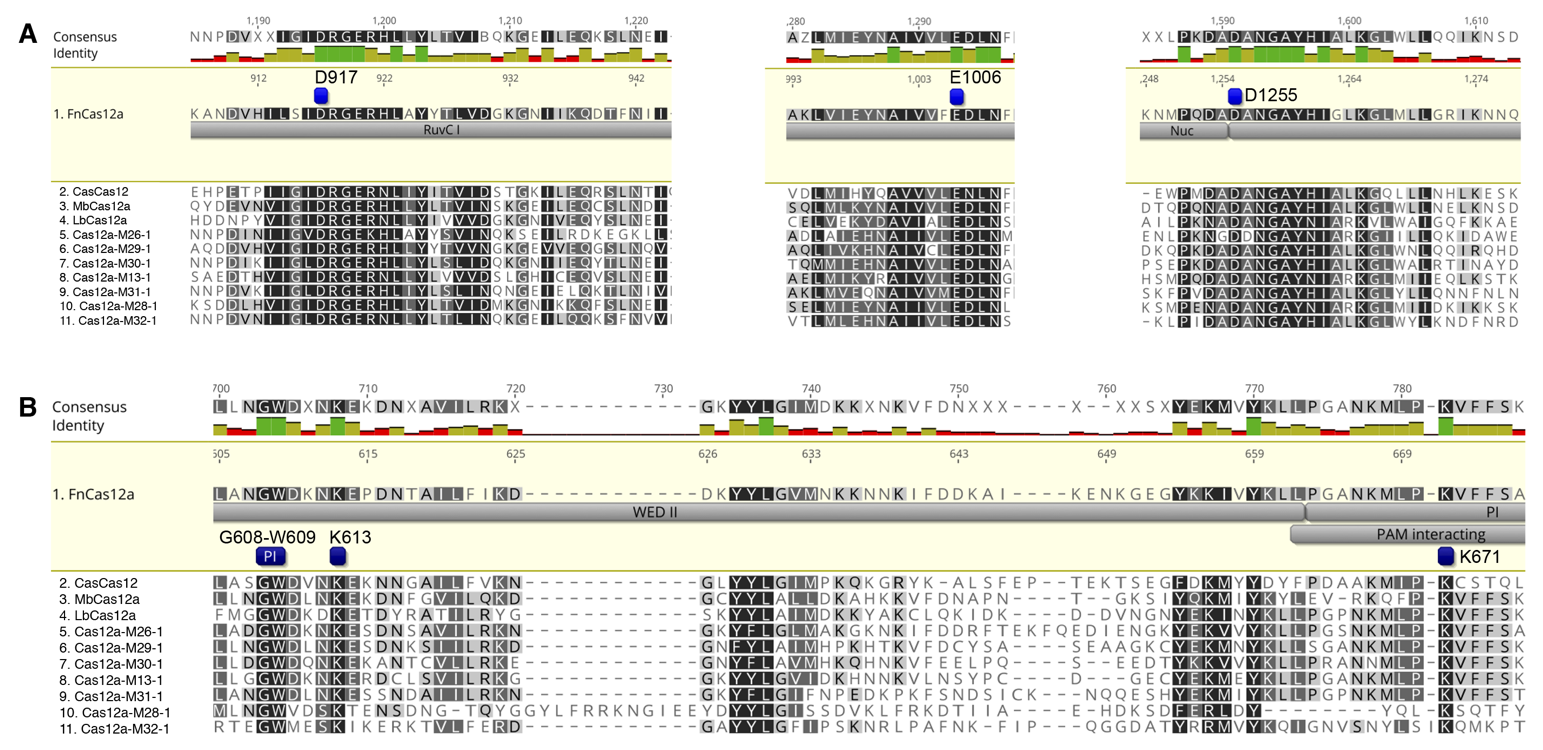

Supplement: Supplemental data [file Supp_Fig2.docx]
