# Supplementary material for: Novel Type V-A CRISPR Effectors Are Active Nucleases with Expanded Targeting Capabilities
Source: CRISPR J. 2020 Dec 17;3(6):454–61. doi: 10.1089/crispr.2020.0043 (PMC7757703; doi:10.1089/crispr.2020.0043)

Supplementary Figure 6. RNP editing profile comparison for Cas12a-M29-1 and AsCas12a. Editing efficiencies at the same target sites are shown for one experiment. Efficiencies were calculated with CRISPResso with window sizes (W) 5, 10, 15, and 20 bp around the predicted cut site. Overall efficiency values are reported in Supplementary Table 4.


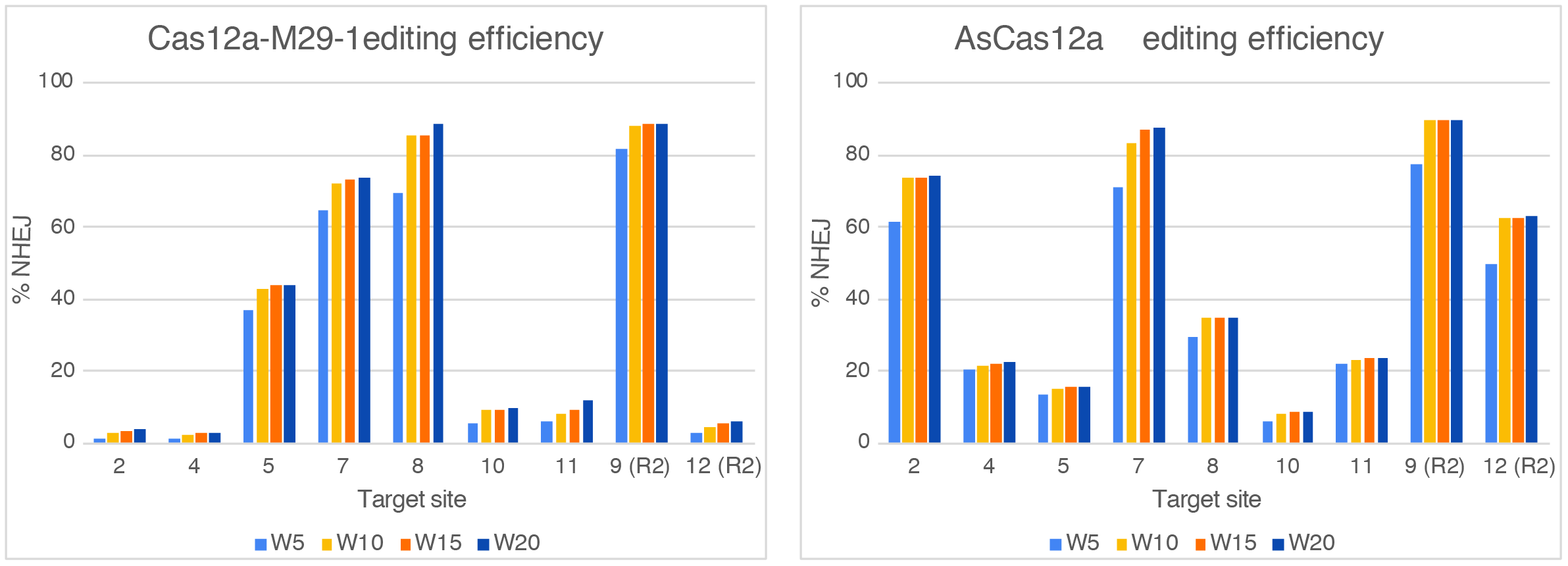

Supplement: Supplemental data [file Supp_Fig6.docx]
